# Supplementary material for: Legacy Effects of Biochar and Compost Addition on Arbuscular Mycorrhizal Fungal Community and Co-Occurrence Network in Black Soil
Source: Microorganisms. 2022 Oct 28;10(11):2137. doi: 10.3390/microorganisms10112137 (PMC9692858; doi:10.3390/microorganisms10112137)
Supplement: Supplementary file 1 [file microorganisms-10-02137-s001.zip › Supplementary Figure.pdf]

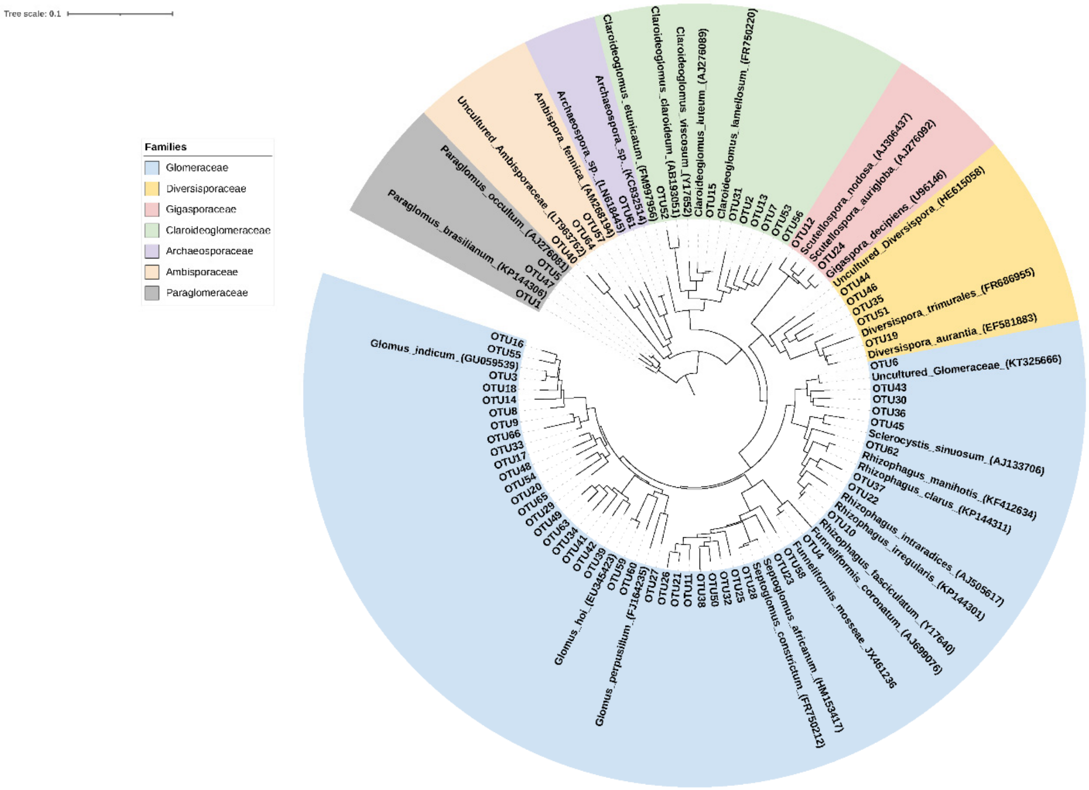

Figure S1 Neighbor-joining tree of arbuscular mycorrhizal (AM) fungi. The 18S rDNA sequences (ca. 334 bp) of AM fungal OTUs obtained in this study and the references downloaded from GenBank were shown concomitantly by the corresponding accession numbers.

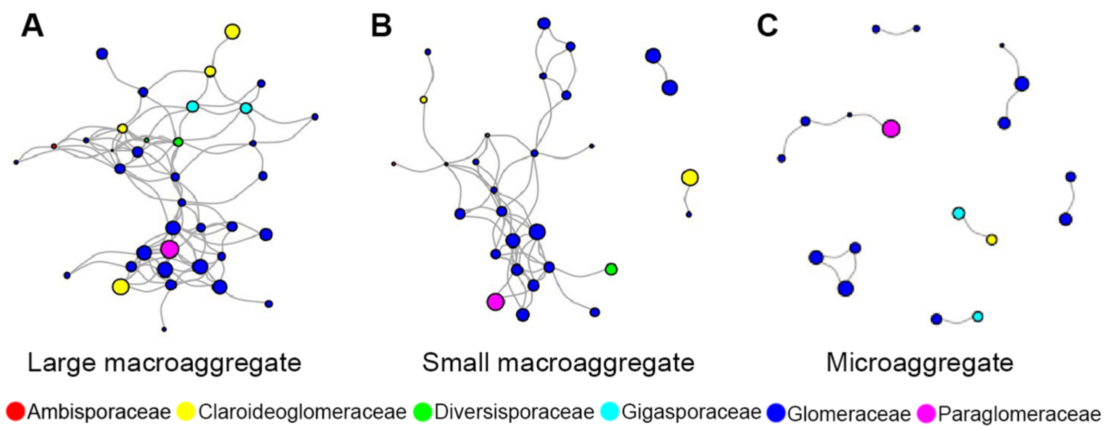

Figure S2 AM fungal co-occurrence networks in large macroaggregate (A), small macroaggregate (B), and microaggregate (C). The size of each node is proportional to its relative abundance, the color of each node represent for the family.
